# Point-prevalence survey of outpatient antibiotic prescription at a tertiary medical center in Sri Lanka: opportunities to improve prescribing practices for respiratory illnesses

**DOI:** 10.1186/s12879-021-05804-6

**Published:** 2021-01-21

**Authors:** Helen L. Zhang, Champica Bodinayake, Gaya B. Wijayaratne, Pasangi Jayatissa, D. L. Bhagya Piyasiri, Ruvini Kurukulasooriya, Tianchen Sheng, Ajith Nagahawatte, Christopher Woods, L. Gayani Tillekeratne

**Affiliations:** 1 Division of Infectious Diseases, University of Pennsylvania Health System, Philadelphia, PA USA; 2 Faculty of Medicine, University of Ruhuna, Galle, Sri Lanka; 3 Duke Global Health Institute, Duke University, Durham, NC USA; 4 Teaching Hospital Karapitiya, Galle, Sri Lanka; 5 Division of Infectious Diseases, Department of Medicine, Duke University School of Medicine, Durham, NC USA

**Keywords:** Antibiotic use, Outpatients, Antibiotic stewardship, Respiratory tract infections, Sri Lanka

## Abstract

**Background:**

Inappropriate antibiotic use is linked to the spread of antimicrobial resistance worldwide, but there are limited systemic data on antibiotic utilization in low- and middle-income countries. The purpose of this study was to evaluate the prevalence and patterns of antibiotic prescription in an ambulatory care setting in Sri Lanka.

**Methods:**

This cross-sectional survey was conducted at the Outpatient Department of a public tertiary medical center in Southern Province, Sri Lanka from February to April 2019. Among consecutive outpatients presenting for care, questionnaires were verbally administered to a systematic random sample to capture information about patient demographics, illness characteristics, and visit outcomes. Prescription data were obtained from the outpatient pharmacy’s electronic prescribing system.

**Results:**

Of 409 surveyed patients, 146 (35.7%) were prescribed an antibiotic. The most frequently prescribed agents were amoxicillin (41 patients, 28.1% of antibiotic recipients) and first-generation cephalosporins (38, 26.0%). Respiratory indications were the most common reason for antibiotic use, comprising 69 (47.3%) of all antibiotic prescriptions. Antibiotics were prescribed for 66.1% of patients presenting with cough and 78.8% of those presenting with rhinorrhea or nasal congestion. Among all antibiotic recipients, 6 (4.1%) underwent diagnostic studies.

**Conclusions:**

A high prevalence of antibiotic prescription was observed, in particular for treatment of respiratory conditions. These data support the need for improved antimicrobial stewardship in the Sri Lankan outpatient setting.

**Supplementary Information:**

The online version contains supplementary material available at 10.1186/s12879-021-05804-6.

## Background

The emergence and spread of antimicrobial resistance (AMR) poses a serious global public health threat. Annually, antimicrobial-resistant infections contribute to an estimated 23,000 deaths in the United States and 25,000 deaths in Europe [1, 2]. While reliable estimates are lacking in low- and middle- income countries (LMICs), morbidity and mortality attributable to AMR are expected to be even greater in these settings due to a higher prevalence of infectious diseases and lesser access to newer-generation antibiotics [3].

Although epidemiologic surveillance of AMR in Sri Lanka has only recently been implemented, available data reveal a high prevalence of infections caused by drug-resistant organisms. In a multi-center study of gram-negative bacterial bloodstream infections, organisms producing extended-spectrum beta-lactamases (ESBLs) accounted for nearly one-quarter of isolated pathogens [4]. Additionally, national surveillance data of urine culture isolates demonstrated resistance to ciprofloxacin and third-generation cephalosporins at proportions exceeding 50% among enteric gram-negative bacteria [5].

Decades of widespread antibiotic use have created selective pressure for the development of AMR, with increasing antibiotic consumption especially pronounced in LMICs [6]. A recent point-prevalence survey of antimicrobial use in Sri Lankan public hospitals revealed that more than one-half of all inpatients and nearly all patients in intensive care wards were receiving antimicrobials at the time of survey; approximately one-third of antimicrobials were deemed to be potentially inappropriate [7]. Systematic data on outpatient antimicrobial prescribing practices in Sri Lanka, however, remain limited. A 2015 multi-center prescription audit performed in Sri Lanka by the World Health Organization South-East Asia Regional Office reported that 45–67% of patients receiving medications from public pharmacies and 21–27% of private pharmacy clients were prescribed an antibiotic [8]. There are no studies that systematically examine outpatient antibiotic prescription in the context of patient-level data in Sri Lanka.

In this study, we determined the point prevalence of antibiotic prescription and assessed demographic and clinical patterns associated with antibiotic prescription among outpatients at a public tertiary healthcare facility in Southern Province, Sri Lanka.

## Methods

### Setting

This cross-sectional study was conducted at a 1600-bed public tertiary teaching hospital located in the Southern Province of Sri Lanka. The hospital provides both inpatient and outpatient services free of cost. Outpatient services include an Outpatient Department (OPD) offering ambulatory acute care services to upwards of 1000 patients per day. The majority of OPD patients are local residents who present by self-referral. The hospital has an on-site outpatient pharmacy to which OPD prescriptions are electronically sent.

### Survey procedures

Pre- and post-visit questionnaires were developed explicitly for this study (Supplementary file 1). Pediatric and adult OPD patients were recruited on consecutive weekdays from February to April 2019 to participate in the study survey. Systematic random sampling was performed by approaching every fifth patient in the OPD waiting queue for study enrollment. Written consent was obtained from patients ≥18 years of age and from the parent/guardian of patients < 18 years, and assent was additionally obtained from patients 12–17 years. After obtaining consent, the pre-visit questionnaire was verbally administered in the local language of Sinhala to obtain information regarding the participant’s demographics, presenting illness, medical co-morbidities, and expectations for the doctor’s visit. Immediately following the doctor’s visit, the post-visit questionnaire was verbally administered to obtain information regarding visit diagnoses, diagnostic tests that were ordered or reviewed during the visit, patient knowledge and perceptions regarding antibiotics, and patient satisfaction with the visit. Prior to being asked questions about antibiotics, all respondents were provided with a definition of antibiotics using lay terminology. For each OPD patient participating in the study, the OPD pharmacy’s electronic prescribing system was queried to obtain information regarding medications prescribed during the visit.

### Data analysis

Survey data were entered into a Research Electronic Data Capture (REDCap) database. Statistical analysis was performed in R version 3.6.3 (Vienna, Austria). Missing responses were omitted from denominators used to calculate simple proportions. Fisher’s exact test with odds ratios and 95% confidence intervals was used to identify demographic and clinical features associated with antibiotic prescription. Two-tailed *p*-values were used, and a *p*-value less than 0.05 was used to define statistical significance.

## Results

### Patient demographics and clinical characteristics

Of 409 total patients enrolled, 88 (21.6%) were children under 18 years of age and 153 (37.5%) were male (Table 1). Median patient age was 38 (interquartile range [IQR] 19–54) years. Chronic medical conditions were reported among 128 (31.4%) patients, with the most common conditions being hypertension (49, 12.0%) and hyperlipidemia (40, 9.8%). The most frequent presenting symptoms were cough (59, 14.5%), musculoskeletal pain (55, 13.5%), and skin rash or boil (34, 8.4%). The median duration of illness at presentation was 7 (IQR 3–30) days. Two hundred thirteen (52.1%) patients had previously visited another healthcare provider for the same illness, with prior OPD evaluations among 114 (27.9%) patients, general practitioner visits among 81 (19.8%) patients, and traditional/Ayurvedic practitioner visits among 9 (2.2%) patients.

**Table 1:** Demographic and clinical characteristics of outpatients attending a public tertiary medical center in southern Sri Lanka (*N* = 409)

Characteristic	n (%)
**Age**	
< 18 years	88 (21.6%)
18–64 years	283 (69.5%)
≥ 65 years	37 (9.1%)
**Gender**	
Male	153 (37.6%)
Female	254 (62.4%)
**Education**^**1**^	
No formal education	25 (6.1%)
≤ 11th grade	254 (62.3%)
12th grade or higher	129 (31.6%)
**Chronic medical conditions**^**2**^	
No chronic medical conditions	280 (68.6%)
Hypertension	49 (12.0%)
Hyperlipidemia	40 (9.8%)
Asthma	39 (9.5%)
Diabetes mellitus	31 (7.6%)
Chronic kidney disease	3 (0.7%)
Chronic liver disease	2 (0.5%)
**Presenting symptom**	
Cough	59 (14.5%)
Musculoskeletal pain	55 (13.5%)
Skin rash or boil	34 (8.4%)
Rhinorrhea or nasal congestion	33 (8.1%)
Fever	31 (7.6%)
Wound	25 (6.1%)
Abdominal pain	15 (3.7%)
Chest pain	11 (2.7%)
Numbness	11 (2.7%)
Headache	10 (2.5%)
Sore throat	10 (2.5%)
Ear pain	7 (1.7%)
Dysuria	6 (1.5%)
Wheezing	5 (1.2%)
Shortness of breath	4 (1.0%)
Fatigue/lethargy	4 (1.0%)
Bleeding	2 (0.5%)
Enlarged lymph node	2 (0.5%)
Medication refill	2 (0.5%)
Other	81 (19.9%)
**Previous evaluations for current illness**^**2**^	
No previous evaluations	196 (47.9%)
General practitioner	81 (19.8%)
Outpatient Department	114 (27.9%)
Traditional/Ayurvedic practitioner	9 (2.2%)
Other healthcare provider	24 (5.9%)
**Previously medicated for current illness**^**2**^	
Non-prescription medication	66 (16.1%)
Prescription medication	176 (43.0%)
Traditional/herbal medicine	60 (14.7%)
No previous medications	138 (33.7%)

^1^Highest attained education of surveyed respondents, comprising adult patients or parents/guardians accompanying pediatric patients

^2^Respondents were counted more than once if multiple categories applied

### Clinical management during encounters

Twenty-three (5.6%) patients reported that they had been given a diagnosis by the physician during their clinical encounter. The majority of patients (379, 92.7%) were not asked to undergo further laboratory or radiographic studies as part of the diagnostic workup. Among patients who received further evaluation, the most frequently ordered studies were full blood count (14, 3.4% of all patients), urinalysis (14, 3.4%), blood glucose testing (4, 1.0%), radiography (2, 0.5%), and electrocardiogram (2, 0.5%). Medications were prescribed for 292 (71.4%) patients, of whom 56 (19.2%) reported that they were informed about the purpose of the prescribed medication(s) by their physician.

### Antibiotic prescribing patterns

**Fig. 1:**
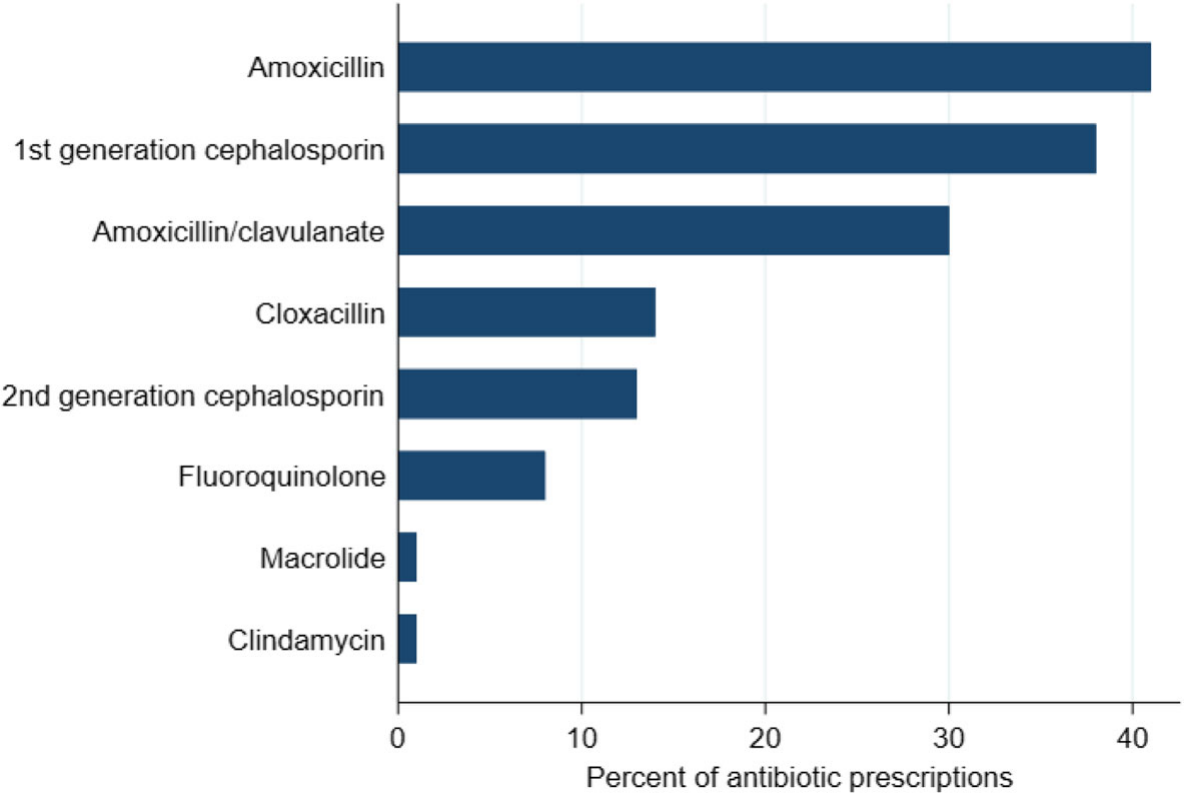
Antibiotics prescribed to outpatients attending a public tertiary medical center in southern Sri Lanka (*N* = 146)

Antibiotics were prescribed for 146 (35.7%) patients, with the most common antibiotics being amoxicillin (41, 28.1% of antibiotic recipients), first-generation cephalosporins (38, 26.0%), and amoxicillin/clavulanate (30, 20.5%). Figure 1 depicts the distribution of prescribed antibiotics. Among patients receiving antibiotics, the most common presenting symptoms were cough (39, 26.7% of antibiotic recipients), rhinorrhea or nasal congestion (26, 17.8%), fever (18, 12.3%), skin rash or boil (9, 6.2%), sore throat (8, 5.5%), and skin wound (7, 4.8%). Amoxicillin and first-generation cephalosporins were most commonly prescribed for cough (36.6% of amoxicillin and 42.1% of first-generation cephalosporin recipients) and rhinorrhea or nasal congestion (22.0% of amoxicillin and 23.7% of first-generation cephalosporin recipients). In contrast, amoxicillin-clavulanate was most frequently prescribed for ear pain (7, 23.3%), fever (4, 13.3%), and cough (4, 13.3%).

Of the 141 antibiotic recipients with a documented duration of therapy, 135 (95.7%) received a 3-day course of antibiotic medication, the standard duration for medications dispensed through the OPD pharmacy. Diagnostic studies were ordered for 6 (4.1%) antibiotic recipients, and study results were not reviewed for any patient prior to receiving an antibiotic prescription. There was a non-significant trend towards increased antibiotic prescription among pediatric patients compared to adult patients (odds ratio [OR] 1.6, 95% confidence interval [CI] 0.9–2.6, *p* = 0.08). Among adults, the likelihood of antibiotic prescription was similar among patients ≥65 years and patients 18–64 years (OR 1.09, 95% CI 0.49–2.34, *p* = 0.85). Adult patients reporting fever (OR 4.7, 95% CI 1.6–15.7, *p* = 0.002) or illness duration less than 7 days (OR 2.3, 95% CI 1.4–3.8, *p* = 0.001) were more likely to receive an antibiotic prescription; those who had prior evaluations for their current illness were less likely to receive an antibiotic (OR 0.5, 95% CI 0.3–0.8, *p* = 0.002; Table 2). Among children, patients with an illness duration less than 7 days (OR 2.6, 95% CI 1.0–7.3, *p* = 0.04) were more likely to receive an antibiotic. Antibiotic prescription did not significantly differ between respondents with ≥12th grade education compared to those with <12th grade education (OR 0.85, 95% CI 0.54–1.35, *p* = 0.51).

**Table 2:** Bivariable analysis of association between outpatient characteristics and antibiotic prescription at a public tertiary medical center in southern Sri Lanka

	Children		Adults	
Characteristic	OR (95% CI)	p-value	OR (95% CI)	p-value
Male gender	1.4 (0.5–3.5)	0.5	0.8 (0.5–1.4)	0.5
Febrile illness	1.8 (0.555.8)	0.3	4.7 (1.6–15.7)	0.002
Presence of medical co-morbidities	1.6 (0.4–7.2)	0.5	0.9 (0.5–1.5)	0.6
Illness duration less than 7 days	2.6 (1.0–7.3)	0.04	2.3 (1.4–3.8)	0.001
Prior medical evaluations for present illness	0.6 (0.2–1.6)	0.3	0.5 (0.3–0.8)	0.002
Prior use of medications for present illness	0.8 (0.3–2.1)	0.7	1.1 (0.6–1.8)	0.9

Abbreviations: *OR* Odds ratio. *CI* Confidence interval

### Antibiotics prescribed for respiratory illnesses

Overall, respiratory symptoms such as cough, rhinorrhea, congestion, wheezing, or shortness of breath comprised the largest indication (69, 47.3%) among patients receiving antibiotic therapy. Among patients with a presenting symptom of cough, 39 (66.1%) were prescribed antibiotics and 4 (7.7%) of these antibiotic recipients reported fever. Among those with rhinorrhea or congestion, 26 (78.8%) received antibiotics and none reported fever. Antibiotics were prescribed to 3 (60%) of 5 patients with a presenting symptom of wheezing and 1 (25%) of 4 patients with a presenting symptom of shortness of breath. The most frequently prescribed antibiotics among patients with respiratory indications were first-generation cephalosporins (26, 37.7%), amoxicillin (26, 37.7%), and second-generation cephalosporins (7, 10.1%). Among all antibiotic recipients with a respiratory presenting symptom, 2 (2.9%) underwent a diagnostic study and none underwent radiography.

### Patient knowledge and expectations regarding antibiotic prescriptions

During pre-visit interviews, 359 (88.2%) respondents expected to be prescribed a medication during their visit and 2 (0.6%) specifically expected an antibiotic prescription. In post-visit interviews, 36 (9.1%) respondents reported knowledge regarding the purpose of antibiotics. After being provided with the definition of an antibiotic, 118 (29.4%) respondents thought that an antibiotic would be helpful for their illness. Among respondents who were prescribed an antibiotic, 32 (22.4%) were aware that they had been prescribed one, and 50 (35.2%) thought that an antibiotic would be helpful for their illness.

### Visit satisfaction

Visit satisfaction was reported among 330 (84.8%) of all respondents. Among all respondents, there was a trend towards increased proportion of visit satisfaction among antibiotic recipients (OR 1.9, 95% CI 1.0–3.9, *p* = 0.05). Visit satisfaction was also greater among antibiotic recipients compared to those receiving prescriptions for other medications (OR 2.3, 95% CI 1.0–5.4, *p* = 0.03). The most frequently reported reasons for visit satisfaction included prescription of medications (190, 57.6%), satisfaction with the physical examination (49, 14.8%), and feeling that the physician listened to the patient (40, 12.1%). Among dissatisfied patients, 3 (5.1%) reported that lack of medication prescriptions contributed to visit dissatisfaction. Ninety (22.4%) patients planned to visit a different provider for their health concern.

## Discussion

This single-center point-prevalence survey demonstrates a high prevalence of outpatient antibiotic prescription at a public tertiary medical center in southern Sri Lanka. Antibiotics were prescribed to approximately one-third of patients in this study, similar to the proportions of 33–54% reported in a 2015 World Health Organization audit of Sri Lankan OPDs as well as antibiotic prescribing figures reported in other South East Asian countries [8, 9]. These findings suggest that the prevalence of antibiotic prescription among outpatient care encounters in Sri Lanka may exceed that observed in some high-income settings; in comparison, an overall antibiotic prescription rate of 12% was previously reported in a nationally representative sample of ambulatory care visits in the United States [10]. However, prescription of broad-spectrum antibiotics was relatively infrequent in our study sample. Narrow-spectrum beta-lactam antibiotics represented the predominant choices for therapy while fluoroquinolone use was less frequent, which was consistent with Sri Lankan national clinical practice guidelines for common syndromes including lower respiratory tract infection and skin and soft tissue infection [11].

Our survey revealed limited knowledge and awareness of antibiotics and resultant low prevalence of expectations for antibiotic prescription among patients, suggesting that antibiotic prescribing behavior may have been driven by healthcare providers rather than by patient demand. These data support previous qualitative data from this outpatient setting showing that patient expectations for antibiotic prescription were uncommon [12]. In contrast, physicians’ perceptions that patients desire antibiotics have been identified as a major driver of antibiotic over-prescribing behavior in multiple studies [12–14]. As such, clinical guidelines and clinician training to improve rational antibiotic prescribing should include strategies to address both actual and perceived patient demand for antibiotics.

Of note, a high proportion of antibiotic prescriptions was given for treatment of respiratory syndromes. Patients with respiratory symptoms comprised nearly one-half of all antibiotic recipients. Conversely, a majority of patients with respiratory illnesses received an antibiotic prescription. These findings parallel previous observations in the inpatient setting in Sri Lanka, where lower respiratory tract infections were the most common indication for antibiotic therapy among hospitalized patients, as well as data from high-income countries showing high rates of outpatient antibiotic prescription for acute respiratory conditions [7, 10, 15]. Interventions targeting prescribing practices for acute respiratory tract infections thus represent a high-yield opportunity for antimicrobial stewardship. It was noteworthy that laboratory or radiographic evaluations to differentiate between upper and lower respiratory tract infections, or between viral and bacterial etiologies, were rarely performed in our study sample. Since the low utilization of diagnostic testing prior to antibiotic prescription may have been influenced by high patient volume and the desire to reduce costs in this public healthcare setting, cost-effectiveness and throughput of testing are important factors to consider when implementing interventions to potentially expand the use of diagnostics. Previous studies in Sri Lanka and other settings have shown that patients with a positive rapid influenza test had lower odds of receiving antibiotics, highlighting a potential role for rapid low-cost diagnostics in reducing antibiotic over-prescription [16, 17].

This study has several limitations. As a point prevalence survey, our data do not capture potential temporal variation in antibiotic prescribing practices in relation to seasonal illnesses such as influenza and dengue. The single center nature of this study precludes the generalization of findings to Sri Lanka due to potential differences between the public and private healthcare sector, primary and secondary/tertiary facilities, and different regions of the country in terms of physician prescribing behavior and patient population. Additionally, all clinical information except for antibiotics prescribed was based on patient self-report and thus may have been subject to reporting bias.

## Conclusions

Antimicrobial stewardship represents a growing priority in Sri Lankan public health as well as the global health arena. Our study highlights a particular need for antimicrobial stewardship in ambulatory settings, for which there is limited experience in LMICs and a dearth of best practice guidelines. The development and scale-up of strategies to provide ongoing surveillance of antibiotic use, increase access to and utilization of diagnostic testing, and reinforce rational antibiotic prescribing practices will be important components in global efforts to control the spread of antimicrobial resistance.

## Supplementary Information

**Additional file 1: Supplementary file 1**. OPD pre- and post-visit questionnaires. English version pre- and post-visit study questionnaires.

## Data Availability

The dataset used during the current study is available from the corresponding author on reasonable request.

## Abbreviations

AMR: Antimicrobial resistance
CI: Confidence interval
ESBL: Extended-spectrum beta-lactamases
IQR: Interquartile range
LMIC: Low- and middle-income country
OPD: Outpatient department
OR: Odds ratio

## References

[1] US Centers for Disease Control and Prevention (2013). Antibiotic resistance threats in the United States.

[2] ECDC/EMEA Joint Working Group. The bacterial challenge: time to react Stockholm, Sweden 2009 [Available from: https://ecdc.europa.eu/sites/portal/files/media/en/publications/Publications/0909_TER_The_Bacterial_Challenge_Time_to_React.pdf.

[3] Laxminarayan R, Duse A, Wattal C, Zaidi AK, Wertheim HF, Sumpradit N (2013). Antibiotic resistance-the need for global solutions. Lancet Infect Dis.

[4] Chandrasiri P, Elwitigala J, Nanayakkara G, Chandrasiri S, Patabendige G, Karunanayaka L (2013). A multi Centre laboratory study of gram negative bacterial blood stream infections in Sri Lanka. Ceylon Med J.

[5] Sri Lanka College of Microbiologists. Report on National Laboratory Based Surveillance on Antibiotic Resistance (NLBSA) of Sri Lanka College of Microbiologists - Data of urine culture isolates 2015 [Available from: http://slmicrobiology.net/download/report-on-2015-data-to-the-web.pdf.

[6] Klein EY, Van Boeckel TP, Martinez EM, Pant S, Gandra S, Levin SA (2018). Global increase and geographic convergence in antibiotic consumption between 2000 and 2015. Proc Natl Acad Sci U S A.

[7] Sheng T, Wijayaratne GB, Dabrera TM, Drew RJ, Nagahawatte A, Bodinayake CK (2019). Point-prevalence study of antimicrobial use in public hospitals in southern Sri Lanka identifies opportunities for improving prescribing practices. Infect Control Hosp Epidemiol.

[8] World Health Organization Regional Office for South-East Asia. Medicines in health care delivery Sri Lanka situational analysis: 16-27 March 2015 2016 [Available from: http://origin.searo.who.int/entity/medicines/sri_lanka_mar_2016.pdf.

[9] Holloway KA, Kotwani A, Batmanabane G, Puri M, Tisocki K (2017). Antibiotic use in South East Asia and policies to promote appropriate use: reports from country situational analyses. BMJ.

[10] Fleming-Dutra KE, Hersh AL, Shapiro DJ, Bartoces M, Enns EA, File TM (2016). Prevalence of inappropriate antibiotic prescriptions among US ambulatory care visits, 2010-2011. JAMA.

[11] Sri Lanka College of Microbiologists. Empirical and prophylactic use of antimicrobials: national guidelines 2016 [Available from: http://slmicrobiology.lk/download/National-Antibiotic-Guidelines-2016-Web.pdf.

[12] Tillekeratne LG, Bodinayake CK, Dabrera T, Nagahawatte A, Arachchi WK, Sooriyaarachchi A (2017). Antibiotic overuse for acute respiratory tract infections in Sri Lanka: a qualitative study of outpatients and their physicians. BMC Fam Pract.

[13] Quet F, Vlieghe E, Leyer C, Buisson Y, Newton PN, Naphayvong P (2015). Antibiotic prescription behaviours in Lao People's Democratic Republic: a knowledge, attitude and practice survey. Bull World Health Organ.

[14] Hassali MA, Kamil TK, Md Yusof FA, Alrasheedy AA, Yusoff ZM, Saleem F (2015). General practitioners' knowledge, attitude and prescribing of antibiotics for upper respiratory tract infections in Selangor, Malaysia: findings and implications. Expert Rev Anti-Infect Ther.

[15] US Centers for Disease Control and Prevention (2017). Antibiotic use in outpatient settings.

[16] Tillekeratne LG, Bodinayake CK, Nagahawatte A, Vidanagama D, Devasiri V, Arachchi WK (2015). Use of rapid influenza testing to reduce antibiotic prescriptions among outpatients with influenza-like illness in southern Sri Lanka. Am J Trop Med Hyg.

[17] Bhavnani D, Phatinawin L, Chantra S, Olsen SJ, Simmerman JM (2007). The influence of rapid influenza diagnostic testing on antibiotic prescribing patterns in rural Thailand. Int J Infect Dis.

