# Supplementary material for: Point-prevalence survey of outpatient antibiotic prescription at a tertiary medical center in Sri Lanka: opportunities to improve prescribing practices for respiratory illnesses
Source: BMC Infect Dis. 2021 Jan 21;21:97. doi: 10.1186/s12879-021-05804-6 (PMC7818802; doi:10.1186/s12879-021-05804-6)
Supplement: Supplementary file 1 — Additional file 1: Supplementary file 1. OPD pre- and post-visit questionnaires. English version pre- and post-visit study questionnaires. [file 12879_2021_5804_MOESM1_ESM.docx]

**PART I. PRIOR TO VISIT**

1. Respondent: 🞎 Patient 🞎 Parent/Guardian

2. Respondent’s highest completed level of education:

🞎 No formal 🞎 <O/L 🞎 O/L 🞎 A/L 🞎 >A/L 🞎 Graduate

3. Patient age: _____ years (If less than 1 year) _____ months

4. Patient gender: 🞎 M 🞎 F

5. Presenting symptoms (check all symptoms):

| 🞎 Abdominal pain  🞎 Bleeding  🞎 Chest pain  🞎 Cough  🞎 Dry 🞎 Productive 🞎 Bloody  🞎 Decreased urination  🞎 Diarrhea  🞎 Fatigue or lethargy  🞎 Fever  🞎 Headache  🞎 Joint pain  🞎 Loss of appetite or eating less | 🞎 Medication refill  🞎 Muscle pain  🞎 Muscle weakness  🞎 Nausea or vomiting  🞎 Painful urination  🞎 Pregnancy  🞎 Runny nose or congestion  🞎 Shortness of breath  🞎 Skin rash or boil  🞎 Sore throat  🞎 Wound  🞎 Other _________________________ |
| --- | --- |

6. Of all the presenting symptoms, which ONE symptom is the primary/most severe complaint? (Circle response above)

7. Duration of present illness: __________ days

8. What does the patient/guardian think caused the present illness? (free response)

___________________________________________________________________________________

9. Previous evaluations for the present illness:

🞎 GP (number of visits): _______

🞎 OPD (number of visits): _______

🞎 Traditional/Ayruvedic practitioner (number of visits): _______

🞎 Other (number of visits): _______

🞎 No previous evaluations

10. Previous medications for the present illness:

🞎 Over-the-counter ________________________________________________

🞎 Prescription ____________________________________________________

🞎 Traditional/herbal ________________________________________________

🞎 No previous medications

11. Chronic medical conditions (check all that apply):

| 🞎 Asthma  🞎 Cancer  🞎 Chronic kidney disease  🞎 Chronic liver disease  🞎 Diabetes mellitus  🞎 Emphysema/COPD | 🞎 Heart failure  🞎 Hypertension  🞎 Ischemic heart disease  🞎 Medication allergy (specify) _____________________  🞎 Other ______________________________________  🞎 No chronic medical conditions |
| --- | --- |
|  |  |

12. What does the patient expect from the current visit? (free response)

____________________________________________________________________________________

13. Does the patient expect medication prescriptions at the current visit?

🞎 Yes, any prescription

🞎 Yes, specific drug/class

🞎 Antibiotic ________________________

🞎 Antihistamine ________________________

🞎 Antihypertensive ________________________

🞎 Antipyretic ________________________

🞎 Hypoglycemic ________________________

🞎 Inhaled bronchodilator ________________________

🞎 Oral steroid ________________________

🞎 Pain medication (non-opioid) ________________________

🞎 Pain medication (opioid) ________________________

🞎 Other ________________________

🞎 No

**PART II. AFTER VISIT**

14. Diagnosis provided at current visit: __________________________________________

🞎 Patient/guardian does not recall

🞎 No diagnosis given

15. Investigations ordered at current visit (check all that apply):

🞎 Blood test(s)

🞎 Full blood count

🞎 Blood sugar

🞎 Basic metabolic panel

🞎 Liver function panel

🞎 Other ___________________________________

🞎 Urinalysis

🞎 Radiographic studies (specify) _______________________________________

🞎 ECG

🞎 Other _______________________________________

🞎 No investigations ordered

16. Did the provider review the results of any investigations during the current visit?

🞎 Yes, from investigations obtained at the current visit

🞎 Yes, from investigations obtained at a prior visit

🞎 No

🞎 Not sure

17. Were medications prescribed during the current visit?

🞎 Yes 🞎 No

18. Did the provider describe the purpose of medications prescribed at current visit?

🞎 Yes 🞎 No 🞎 No medications were prescribed

19. Does the patient know what an antibiotic is?

🞎 Yes, describe: __________________________________________________________

🞎 No

*At this time, please describe that antibiotics are medicines used to treat bacterial infections.*

20. Does the patient/guardian think that antibiotics were prescribed during this visit?

🞎 Yes 🞎 No 🞎 Not sure 🞎 No medications were prescribed

21. Does the patient/guardian think that antibiotics would be helpful for the present illness?

🞎 Yes 🞎 No 🞎 Not sure

22. Was the patient/guardian satisfied with the current visit?

🞎 Yes 🞎 No

23. Why was the patient satisfied or dissatisfied? (check all that apply)

| 🞎 Doctor was kind  🞎 Doctor listened/asked questions  🞎 Doctor took time  🞎 Doctor did a good examination  🞎 Doctor ordered investigations  🞎 Doctor prescribed medications  🞎 Wait was short  🞎 Other (specify) ______________ | 🞎 Doctor was not kind  🞎 Doctor did not listen/ask questions  🞎 Doctor did not take time  🞎 Doctor did a poor examination  🞎 Doctor did not order investigations  🞎 Doctor did not prescribe medications  🞎 Wait was long |
| --- | --- |

24. Does the patient/guardian plan to visit a different healthcare provider regarding the present illness?

🞎 Yes (specify) __________________________________________

🞎 No

🞎 Not sure

🞎 Only if they do not get better

25. Additional comments (free response):

**PART III. PHARMACY RECORD REVIEW**

26. Number of medications prescribed at current visit: ___________

27. Receiving pharmacy: 🞎 OPD pharmacy 🞎 Outside pharmacy

28. Systemic antibiotics prescribed at current visit:

🞎 Amoxicillin

🞎 Amoxicillin/clavulanic acid

🞎 1^st^ generation cephalosporin (e.g. cephalexin)

🞎 2^nd^ generation cephalosporin (e.g. cefuroxime, cefaclor)

🞎 3^rd^ generation cephalosporin (e.g. cefdinir, cefixime)

🞎 Clindamycin

🞎 Doxycycline or tetracycline

🞎 Fluoroquinolone (ciprofloxacin, moxifloxacin, levofloxacin, norfloxacin)

🞎 Macrolide (azithromycin, clarithromycin)

🞎 Metronidazole

🞎 Nitrofurantoin

🞎 Penicillin

🞎 Trimethoprim/sulfamethoxazole

🞎 Other ________________________________

🞎 No antibiotics prescribed

29. (If antibiotics prescribed) Duration of antibiotics: ________ days

30. Oral steroids prescribed at current visit:

🞎 Dexamethasone

🞎 Hydrocortisone

🞎 Prednisone or prednisolone

🞎 Other ________________________________

🞎 No steroids prescribed

31. (If oral steroids prescribed) Duration of oral steroids: ________ days

32. Topical antimicrobials prescribed at current visit:

🞎 Topical antibiotic

🞎 Topical antifungal

🞎 No topical antimicrobials prescribed

33. Topical steroids prescribed at current visit

🞎 Yes

🞎 No
